# The relationship between various measures of obesity and arterial stiffness in morbidly obese patients

**DOI:** 10.1186/1471-2261-11-7

**Published:** 2011-02-01

**Authors:** N Nordstrand, E Gjevestad, KN Dinh, D Hofsø, J Røislien, E Saltvedt, I Os, J Hjelmesæth

**Affiliations:** 1 Morbid Obesity Centre, Vestfold Hospital Trust, Tønsberg, Norway; 2 Hospital for Rehabilitation, Department of Physical Medicine, Stavern, Norway; 3 Oslo University Hospital, Department of Biostatistics, Institute of Basic Medical Sciences, Rikshospitalet, Norway; 4 Oslo University Hospital, Department of Nephrology and Hypertension, Ullevål, Norway

## Abstract

**Background:**

Obesity is associated with increased risk of cardiovascular disease. Arterial stiffness assessed by carotid femoral pulse wave velocity (PWV) is an independent predictor of cardiovascular morbidity and mortality. We aimed to investigate how various measures of body composition affect arterial stiffness.

**Methods:**

This is an analysis of cross-sectional baseline data from a controlled clinical trial addressing changes in arterial stiffness after either surgery or lifestyle intervention in a population of morbidly obese patients. High-fidelity applanation tonometry (Millar^®^, Sphygmocor^®^) was used to measure pulse wave velocity (PWV). Carotid femoral PWV is a direct measure of arterial stiffness and is considered to be the gold standard method. The Inbody 720 Body Composition Analyzer was used for bioelectrical impedance analysis (BIA). Spearman's correlation, independent samples *t*-test, chi-square tests, Fisher's exact test and multiple linear regression analyses were used as statistical methods.

**Results:**

A total of 133 patients (79 women), with a mean (SD) age of 43 (11) years were included in the study. Men had a significantly higher prevalence of obesity related comorbidities and significantly higher PWV, 9.1 (2.0) m/s vs. 8.1 (1.8) m/s, p = 0.003, than women. In the female group, PWV was positively correlated with WC, WHtR, BMI and visceral fat area. In the male group, PWV was negatively correlated with BMI. Multiple linear regression analysis showed that increasing BMI, WC, WHtR, visceral fat area and fat mass were independently associated with higher PWV in women, but not in men, after adjustment for age, hypertension and type 2 diabetes.

**Conclusion:**

Most measures of general and abdominal obesity were predictors of arterial stiffness in female morbidly obese patients.

**Trial registration:**

ClinicalTrials.gov Identifier NCT00626964

## Background

The prevalence of obesity is rising globally, independent of ethnicity, race and age, and is associated with increased mortality and morbidity [1,2]. Obesity rates in Scandinavia represent no exception [3-5]. Importantly, the prevalence of morbid obesity, defined as BMI ≥ 40.0 kg/m^2 ^or BMI 35.0 -39.9 kg/m^2 ^and an obesity-related comorbidity, is increasing at an even steeper rate [6]. Two percent of the Norwegian population[7] can be classified as morbidly obese.

Several lines of evidence indicate that the distribution of fat is a major determinant of cardiovascular risk in both normalweight, overweight and moderately obese subjects [8-10], although measures of general obesity and physiological differences between genders must also be considered [11-13]. Only a few studies in morbidly obese subjects have compared the effect of overall and central obesity on cardiovascular risks and the results have been inconsistent both within, and between genders [14-16]. Other studies have reported an increased risk of death in persons with BMI > 35 kg/m^2 ^[17,18], but the increase in mortality has been linked to comorbidities rather than the obesity [19] suggesting that morbid obesity without comorbidities is not a cardiovascular risk factor regardless of fat distribution.

Nevertheless, obesity is a major modifiable risk factor for coronary artery disease (CAD) that imparts a degree of cardiovascular risk similar to that associated with hypertension, hyperlipidemia, smoking and sedentary lifestyle [20]. Accordingly, it seems to be a paradox that the increasing obesity rates coincide with a decreased risk of cardiovascular death [21]. The latency time for developing cardiovascular disorders may, however, be several decades [22,23]. Thus, early risk markers are needed to identify obese subjects at risk.

Arterial stiffness assessed by pulse wave velocity (PWV) is an independent predictor of cardiovascular morbidity and mortality [24,25], and PWV is increasingly recognized as a valid surrogate endpoint of cardiovascular disease [26,27].

The main objective of this study was to examine the possible relationship between various measures of body composition and arterial stiffness PWV. We hypothesized that in morbidly obese subjects abdominal obesity is a better predictor of aortic stiffness than general obesity.

## Methods

### Study design

This is an analysis of cross-sectional baseline data from a non-randomized clinical trial comparing the effects of a comprehensive lifestyle modification program and bariatric surgery on arterial stiffness in morbidly obese patients. (http://ClinicalTrials.gov Identifier NCT00626964).

### Setting

The study was performed at a tertiary care centre (the Morbid Obesity Centre, Vestfold Hospital Trust, Tønsberg, Norway) between February 2008 and January 2010. Before inclusion patients were either assigned to a comprehensive lifestyle modification program at the Hospital for Rehabilitation, Stavern, or to bariatric surgery at the Morbid Obesity Centre.

### Participants and study size

All participants were recruited from our tertiary care centre and had to reside within 100 km of either the hospital for rehabilitation or our clinic. The patients in the intensive lifestyle intervention group were all selected from patients that had signed up for participation in a standardized health promoting and weight reductive program at the hospital for rehabilitation. Participants in the surgery group were selected from patients preparing for bariatric surgery at our hospital. The decision regarding the type of intervention was made prior to the inclusion to our study and was not a part of our protocol. Patients with one of the following conditions or diseases were excluded; uncompensated heart failure, cardiac arrhythmias, unstable angina, end-stage renal disease, known bleeding disturbances, serious psychiatric disorders, serious eating disorders, cardiac pacemakers, intra-cardiac devices, cerebrovascular event or a myocardial infarction within the last six months. The inclusion period started February 2008 and ended February 2010. Initially, 148 patients accepted our invitation to participate in the study, 15 patients withdrew their consent before the study started, leaving a total of 133 patients in the present analysis. The study was approved by the regional ethics committee of the Southern Norway Regional Health Authority, and was performed in accordance with the Declaration of Helsinki [28]. Written informed consent was provided by all participants.

### Variables

The main outcome variable was arterial stiffness measured by PWV. The main explanatory variables were various measures and estimates of body composition, including anthropometric characteristics (WC, WHR, WHtR, BMI), data from bioelectrical impedance analysis (BIA) [visceral fat area (cm2), fat mass (kg) and fat free mass (kg)]. Potential confounders adjusted for were age, type 2 diabetes and arterial hypertension.

### Data sources and measurements

All participants underwent a medical examination performed by a physician and a trained nurse. Blood was collected by venipuncture following an overnight fast on the day of medical examination. Weight and height were measured with patients wearing light clothing and no shoes. BMI was calculated as weight in kilograms divided by the square of the height in meters. WC (cm) was measured midway between the 12th rib and the iliac crest. Blood pressure was measured after five minutes of rest using an electronic auscultatory blood pressure recorder with an appropriately sized cuff based on the measurement of arm circumference (Dinamap^®^, ProCare Series, G.E. Medical Systems) with the patient sitting in the upright position. Three measurements were recorded, and the average of the second and third measurement was recorded and used in the analysis. Bioelectrical impedance measures were collected using the Inbody 720, Body Composition Analyzer, Biospace Co. Ltd. Arterial hypertension was defined by either, a systolic blood pressure ≥ 140 mmHg, diastolic blood pressure ≥ 90 mmHg or the use of antihypertensive medication. Ischemic heart disease was defined as a history of stable coronary artery disease, percutaneous coronary intervention, coronary artery bypass graft surgery or myocardial infarction. The statistical analysis was performed using SPSS 16.0 (SPSS Inc., Chicago, IL).

### Pulse wave velocity

The measurement of PWV is generally accepted as the simplest, non-invasive, robust, and reproducible method to determine arterial stiffness and is considered to be the gold standard method [29-32]. ]. The Sphygmocor system (Artcor, Sidney, Australia) and a single high-fidelity applanation tonometer (Millar^®^) were used to measure PWV. Pulse waves were obtained sequentially from the carotid and femoral artery. The PWV was calculated from the transit time and the distance between these two arterial sites, determined in relation to the R-wave of the ECG, with patients lying in a horizontal position. Three complete sets of data were sampled and the average value was used as result. Bioelectrical impedance analysis was obtained using Inbody 720, Body Composition Analyzer, Biospace Co. Ltd. Each patient was undressed except for underwear, and placed in an upright position on the body composition analyzer. All jewelry and wristwatches were removed before the analyzer started recording. The InBody 720 uses the segmental BIA method in order to examine the body as five cylinders (four limbs and a trunk), and measures impedance in these parts separately. It also uses electrical current at multi-frequency (5, 50, 250, 500 and 1000 kHz) in order to directly measure the amount of extracellular and intracellular water. Homeostasis Model Assessment Insulin Resistance (HOMA- IR) was calculated as ([fasting serum glucose (mmol/l) × fasting serum insulin (pmol/l)]/135 [33]. Low-density lipoprotein cholesterol (LDL) concentrations were estimated by the Friedewald equation: LDLcholesterol = Total-cholesterol - HDL-cholesterol - (0.45 × triglycerides) [34]. S-LDL cholesterol was not calculated if S-triglycerides were < 0.2 mmol/L or ≥ 5 mmol/L.

### Laboratory analyses

Analyses of serum glucose and blood lipids were performed using dry reagent slide technology on the Vitros FS 5.1 (Ortho-Clinical Diagnostics, New York, USA). Glycated hemoglobin (HbA1c) was analyzed using high performance liquid chromatography on Tosoh HLC-723 G7 (Tosoh Corporation, Tokyo, Japan). Sera for analysis of insulin were stored at - 20°C and analyzed within one week of blood sampling (Linco Research Inc, St. Charles, MO). Immunoturbidimetric method was used for determinations of apolipoprotein A-1 and apolipoprotein B (Modular, Roche Diagnostic GmbH).

### Statistical methods

Data are given as mean (SD) or n (%). Skewed data were ln-transformed to approximate normality before statistical analyses (HOMA-IR, S-f-glucose, S-f-insulin and S-triglycerides). Spearman's rank correlation was used to assess the bivariate association between the outcome and each of the exposure variables. Gender differences were analyzed using independent samples *t*-test for continuous data, whilst Fisher's exact tests were used for categorical data. The multiple linear regression analyses included the various measures of body composition, age, hypertension and type 2 diabetes as independent variables, and aortic PWV as dependent variable. Significant interactions between gender and BMI (p < 0.001), WC (p = 0.019) and WHtR (p < 0.001) made it appropriate to perform gender specific linear regression analyses. Semi-partial (part) correlation coefficients were squared in order to calculate the percentage of total variance in the dependent variable explained by a given independent variable.

## Results

A total of 133 morbidly obese persons (79 women), with a mean (SD) age of 43 (11) years, were included in the study. One hundred and six patients (59 women) had a BMI ≥ 40 kg/m^2^. Thirty one patients (24 women) were morbidly obese based on BMI alone (no comorbidity and BMI ≥ 40 kg/m^2^), whilst 27 patients (18 women) had a BMI between 35 and 40 kg/m^2^. Demographic characteristics, body composition and cardiovascular risk factors according to gender are shown in Table 1. The male patients had a significantly higher prevalence of hypertension, type 2 diabetes and ischemic heart disease, as well as higher levels of fasting insulin, glucose and HbA1c than the female patients. Conversely, men had lower levels of total cholesterol, LDL-cholesterol and HDL-cholesterol than women. The male patients had higher PWV than the female patients, [mean (SD) 9.1 (2.0) m/s vs. 8.1 (1.8) m/s, p = 0.003].

**Table 1:** Demographic characteristics, body composition, metabolic parameters and medications of 133 morbidly obese patients

*Variable*	*Total (n = 133)*	*Male (n = 54)*	*Female (n = 79)*	*P-value*
Age (years)	43 (11)	45 (11)	41 (10)	0.107
Hypertension* (yes/no)	90 (68%)	44 (81%)	46 (58%)	0.005
Type 2 diabetes (yes/no)	38 (29%)	25 (46%)	13 (17%)	<0.001
Ischemic heart disease (yes/no)	7 (5.3%)	6 (11.1%)	1 (1.3%)	0.018
Current smoker (yes/no)	20 (15%)	6 (11%)	14 (18%)	0.340
Weight (kg)	132 (22)	146 (23)	121 (16)	<0.001
*Various measures of body composition*				
Waist-to-hip ratio	0.99 (0.10)	1.17 (0.07)	0.94 (0.07)	<0.001
Waist-to-height ratio	75.3 (6.4)	79.2 (6.2)	74.4 (5.9)	<0.001
Waist circumference (cm)	131 (14)	142 (12)	124 (10)	<0.001
BMI (kg/m^2^)	44.5 (5.6)	46.1 (6.3)	43.5 (4.8)	0.008
Fat mass (kg)	63 (13)	66 (16)	61 (11)	0.055
Fat free mass (kg)	69 (13)	81 (10)	61 (7)	<0.001
Visceral fat area (cm^2 ^)	242 (48)	262 (55)	228 (37)	<0.001
*Metabolic variables*				
apoB/apoA-1 ratio	0.7 (0.2)	0.7 (0.2)	0.7 (0.2)	0.550
Fasting insulin (pmol/L)	100 (85)	125 (120)	87 (49)	0.048
HOMA-IR†	4.6 (4.4)	6.1 (6.3)	3.5 (2.0)	0.002
Fasting glucose (mmol/l)	6.0 (2.2)	6.5 (2.7)	5.4 (1.1)	0.002
B-HbA1c (%)	6.2 (1.3)	6.5 (1.6)	5.7 (0.7)	0.001
Pulse pressure (mmHg)	62 (16)	63 (15)	61 (17)	0.471
*Medication*				
β-blocker (yes/no)	13 (10%)	8 (15%)	5 (6%)	0.134
Calsium-channel blocker (yes/no)	17 (13%)	10 (19%)	7 (9%)	0.125
Inhibitors of the RAA-system (yes/no)	37 (28%)	23 (43%)	14 (18%)	0.003
Statins (yes/no)	18 (14%)	13 (24%)	5 (6%)	0.008

Data are given as number (%) or mean (SD). *Hypertension is defined by the patient receiving antihypertensive medication or having systolic blood pressure ≥ 140 mmHg or diastolic blood pressure ≥ 90 mmHg. † Homeostasis Model Assessment Insulin Resistance

### Women

**Figure 1:**
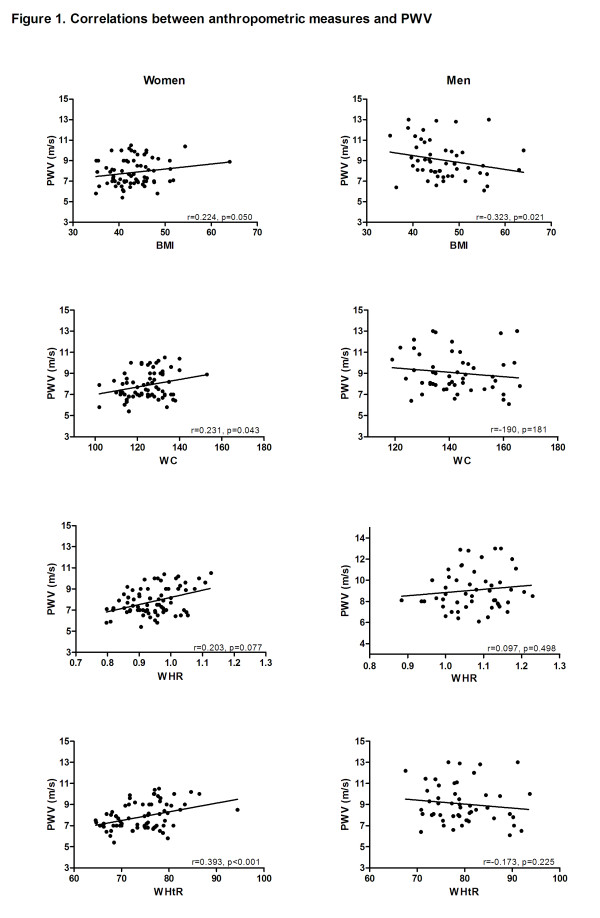
**Correlations between anthropometric measures and PWV**. The scatterplots show the relationship between anthropometric measures and PWV.

In the female group, PWV was positively correlated with BMI, WC and WHtR (Figure 1). Table 2 shows that PWV was significantly correlated with visceral fat area, fasting glucose, HbA1c and the usage of β-blockers and calcium-channel blockers. Multiple linear regression analysis showed that BMI, WC, WHtR, visceral fat area and fat mass, but not WHR, were significantly associated with PWV after adjustment for age, hypertension and type 2 diabetes (table 3). Based on part correlation BMI explained 14% of the variation in PWV, with WHtR explaining 12%, fat mass 8%, visceral fat area 8% and WC 6%.

**Table 2:** Correlations between PWV, body composition, metabolic parameters and medications

Independent variables	Male (r)	Female (r)
Age	0.61†	0.52†
Hypertension‡	0.19	0.59†
Type 2 diabetes (yes/no)	0.46†	0.18
Visceral fat area (cm^2^)	-0.18	0.25*
Fat mass (kg)	-0.26	0.06
Fat free mass (kg)	-0.15	-0.04
apo-B/apoA-1 ratio	-0.29	0.10
Fasting insulin (mmol/L)	0.03	0.09
HOMA IR§	0.10	0.13
Fasting glucose (mmol/L)	0.35*	0.24*
HbA1c (%)	0.27	0.31*
β-blocker (yes/no)	0.12	0.39†
Calsium-channel blocker (yes/no)	0.36*	0.24*
Inhibitors of the RAA-system (yes/no)	0.29*	0.14
Statins (yes/no)	0.39*	0.22

*p < 0.05. †p < 0.001. ‡ Hypertension is defined by the patient receiving antihypertensive medication or having systolic bloodpressure ≥ 140 mmHg or diastoic blood pressure ≥ 90 mmHg. §Homeostasis Model Assessment Insulin Resistance

**Table 3:** Independent predictors of PWV after adjustment for age, hypertension and type 2 diabetes.

Body composition	Β	95% CI	P	R Square
*Female*				
Body mass index (kg/m^2^)	0.129	0.068 to 0.190	<0.001	0.42
Waist circumference (cm)	0.047	0.010 to 0.083	0.013	0.33
Waist-to-hip ratio	-1.297	-6.109 to 3.522	0.594	0.28
Waist-to-height ratio	0.108	0.052 to 0.164	<0.001	0.40
Visceral fat area (cm^2^)	0.014	0.005 to 0.024	0.004	0.35
Fat mass (kg)	0.047	0.015 to 0.080	0.004	0.35
Fat free mass (kg)	0.009	-0.046 to 0.065	0.736	0.27
*Male*				
Body mass index (kg/m^2^)	-0.009	-0.083 to 0.065	0.806	0.47
Waist circumference (cm)	0.014	-0.023 to 0.052	0.440	0.48
Waist-to-hip ratio	1.933	-3.707 to 7.574	0.494	0.48
Waist-to-height ratio	0.021	-0.053 to 0.094	0.575	0.48
Visceral fat area (cm^2^)	0.003	-0.005 to 0.011	0.470	0.49
Fat mass (kg)	0.005	-0.024 to 0.035	0.719	0.48
Fat free mass (kg)	0.008	-0.033 to 0.050	0.693	0.48

Multiple linear regression analysis

### Men

In the male group, PWV showed a significant negative correlation with BMI (Figure 1). The use of calcium-channel blockers, inhibitors of the RAA-system, statins and fasting glucose were positively correlated with PWV (Table 2). BMI showed a negative association with age, r = -0.449, p = 0.001. Multiple linear regression analysis (Table 3) did not demonstrate any significant association between measures of body composition and PWV after adjustment for age, hypertension and type 2 diabetes.

## Discussion

### Key results

Our results indicate that measures of both general obesity and abdominal obesity are associated with PWV in morbidly obese women, but not in men. Thus, we could not confirm our initial hypothesis that abdominal obesity was a better predictor of aortic stiffness than general obesity.

### Comparison with previous studies

Our results support the findings of a previous study of normal weight and less obese European Caucasians which demonstrated that PWV increased with higher BMI in middle-aged and older women, but not in men [35]. Further, we are able to extend these findings to be valid in morbidly obese women. Partly in contrast with our findings, WC, but not BMI, was significantly associated with PWV in a cross-sectional study of middle aged, apparently healthy subjects [36]. In addition, no significant correlation between BMI and PWV was revealed in a recent prospective study of 29 morbidly obese subjects [37]. These discrepancies might have several explanations, including the low percentage of obese participants in the former study and the low number of participants in the latter. On the other hand, a study of less obese African-Americans and whites demonstrated that increased BMI, WC, and WHR were associated with higher PWV in both men and women [38]. Finally, baseline data from a longitudinal study of 50 (12 men) healthy obese patients (BMI > 30 kg/m2) [39] showed that BMI and total fat mass, but not visceral fat mass, assessed by magnetic resonance imaging, were significantly associated with PWV in both genders. The latter is in opposition to our finding of a significant association between visceral fat area assessed by BIA and PWV in women. This difference in results might partly be explained by the higher degree of obesity in our study population and the larger proportion of metabolic disorders.

### Gender differences

It is well known that for any given BMI, men have a larger amount of visceral adipose tissue and a higher risk of cardiovascular disease than women [13]. Our results are in agreement with this. Accordingly, our findings are discussed separately in men and women [40]. In the present study we observed an apparent lack of association between obesity and PWV among male patients in the multivariate analysis (Table 3). This might partly be explained by a significantly higher prevalence of comorbidities and conditions known to increase PWV; such as hypertension, type 2 diabetes, ischemic heart disease and insulin resistance in our male population. Conversely, a weak negative correlation between BMI and PWV was shown in the univariate analysis of male subjects (Figure 1). This surprising finding might partly be explained by the coexistence of a significant negative correlation between age and BMI and the moderate positive correlation between age and PWV in our study. Finally, we cannot exclude the possibility that the number of male patients was too low to reveal a possible association between obesity and PWV.

### Body Composition

The associations between various measures of overall and abdominal obesity and PWV in women were comparable in the present study. However, the amount of fat tissue associated with morbid obesity is likely to cause an accumulation of both visceral and subcutaneous fat. The effect of this can be a loss of superiority of one anthropometric measure of obesity to the other. Another area of concern is the relatively low reliability of the different anthropometric measures in this population[41]. This could imply that the correlations are underestimated and thus should be interpreted with caution. In addition, and in accordance with previous studies [9,42], the anthropometric indexes taking height into account (BMI and WHtR) seemed to explain a larger part of the variation in PWV than WC, visceral fat area and fat mass. Accordingly, the usage of WC may underestimate the relative amount of abdominal fat in short subjects and overestimate it in tall subjects and consequently be misleading.

It is difficult to explain the lack of association between WHR and PWV in our study. This finding is in opposition to several other studies designed to explore the predictive value of various measures of obesity in relation to cardiovascular risk factors [43]. The detrimental effects of abdominal obesity may mitigate against the possible protective effect of subcutaneous fat located at the hip, despite only slightly increased WHR in morbidly obese patients. The low reliability of WHR as compared with WC in a morbidly obese population is also a factor that should be considered [41].

### Other factors related to arterial stiffness

In accordance with a recent study [44] demonstrating a significant association between fasting glucose and PWV in middle aged overweight and obese subjects, we found a significant positive correlation between fasting glucose and PWV in both men and women. In women, but not in men, we also found a positive correlation between HbA1c and PWV. Conversely, we could not demonstrate any association between HOMA-IR and PWV as shown in the previous study [44]. An elevated glucose level can cause a non-enzymatic glycation of matrix proteins and result in the accumulation of glycation end-products within the arterial wall and subsequent arterial stiffness [45].

### Limitations and strengths

The major strengths of the present study are that arterial stiffness is a well validated risk marker of cardiovascular morbidity and mortality, as well as that PWV is considered to be the "gold standard" in assessing arterial stiffness. In terms of weaknesses, the cross-sectional design of the study necessarily implies that we cannot establish a cause-effect relationship. All but one participant, were white, and we are thus unable to generalize our results to non-white populations. It should also be noted that BIA is not considered to be a "gold standard" method. BIA has a tendency to underestimate the amount of fat tissue in morbidly obese patients when compared to hydrostatic weighing and dual-energy X-ray absorptiometry [46-48], although this is minimized by the usage of the segmental BIA method and multifrequency technology in the present study.

## Conclusion

Most measures of general and abdominal obesity were associated with arterial stiffness in morbidly obese women.

## Competing interests

The authors declare that they have no competing interests.

## Authors' contributions

NN and JH: contributed to the study design and organized the study; NN analyzed the data and wrote the manuscript; NN, EG and KN: performed the technical analysis of arterial stiffness and were involved in drafting the manuscript and revised it critically for important intellectual content. JR: reviewed the statistical analyses and revised the manuscript critically for important intellectual content; JH, DH, IO, and ES: contributed to interpretation of data, were involved in drafting the manuscript and revised it critically for important intellectual content. All authors read and approved the final manuscript

## Pre-publication history

The pre-publication history for this paper can be accessed here:

http://www.biomedcentral.com/1471-2261/11/7/prepub

## References

[B1] Lobstein T Jackson-Leach R Child overweight and obesity in the USA: prevalence rates according to IOTF definitions International Journal of Pediatric Obesity 2007 2 62 64 10.1080/17477160601103948 17763012

[B2] Prentice AM The emerging epidemic of obesity in developing countries. [Review] [54 refs] International Journal of Epidemiology 2006 35 93 99 10.1093/ije/dyi272 16326822

[B3] Droyvold WB Nilsen TI Kruger O Holmen TL Krokstad S Midthjell K Change in height, weight and body mass index: Longitudinal data from the HUNT Study in Norway International Journal of Obesity 2006 30 935 939 10.1038/sj.ijo.0803178 16418765

[B4] Neovius M Janson A Rossner S Prevalence of obesity in Sweden. [Review] [15 refs] Obesity Reviews 2006 7 1 3 10.1111/j.1467-789x.2006.00190.x 16436097

[B5] Bendixen H Holst C Sorensen TI Raben A Bartels EM Astrup A Major increase in prevalence of overweight and obesity between 1987 and 2001 among Danish adults Obesity Research 2004 12 1464 1472 10.1038/oby.2004.183 15483211

[B6] Sturm R Increases in morbid obesity in the USA: 2000-2005 Public Health 2007 121 492 496 17399752 10.1016/j.puhe.2007.01.006 PMC2864630

[B7] Graff-Iversen S Jenum AK Grøtvedt L Bakken B Selmer RM Søgard AJ Risikofaktorer for hjerteinfarkt, hjerneslag og diabetes i Norge Tidsskrift for Den Norske Laegeforening 2007 127 2537 2541 17925823

[B8] Larsson B Svardsudd K Welin L Wilhelmsen L Bjorntorp P Tibblin G Abdominal adipose tissue distribution, obesity, and risk of cardiovascular disease and death: 13 year follow up of participants in the study of men born in 1913 British Medical Journal Clinical Research Ed 1984 288 1401 1404 6426576 10.1136/bmj.288.6428.1401 PMC1441047

[B9] Schneider HJ Friedrich N Klotsche J Pieper L Nauck M John U The predictive value of different measures of obesity for incident cardiovascular events and mortality Journal of Clinical Endocrinology & Metabolism 2010 95 1777 1785 10.1210/jc.2009-1584 20130075

[B10] Yusuf S Hawken S Ounpuu S Bautista L Franzosi MG Commerford P Obesity and the risk of myocardial infarction in 27,000 participants from 52 countries: a case-control study Lancet 2005 366 1640 1649 10.1016/S0140-6736(05)67663-5 16271645

[B11] van D Kromhout D Geleijnse JM Boer JM Verschuren WM Body mass index and waist circumference predict both 10-year nonfatal and fatal cardiovascular disease risk: study conducted in 20,000 Dutch men and women aged 20-65 years European Journal of Cardiovascular Prevention & Rehabilitation 2009 16 729 734 10.1097/HJR.0b013e328331dfc0 19809330

[B12] Wang W Zhao D Sun JY Liu J Liu J Qin LP [Predictive value of combined measurements of body mass index and waist circumference for the risk of cardiovascular disease]. [Chinese] Chung-Hua Hsin Hsueh Kuan Ping Tsa Chih [Chinese Journal of Cardiology] 2008 36 655 658 19100099

[B13] Geer EB Shen W Gender differences in insulin resistance, body composition, and energy balance. [Review] [156 refs] Gender Medicine 2009 6 Suppl 75 10.1016/j.genm.2009.02.002 PMC2908522 19318219

[B14] Drapeau V Lemieux I Richard D Bergeron J Tremblay A Biron S Waist circumference is useless to assess the prevalence of metabolic abnormalities in severely obese women Obesity Surgery 2007 17 905 909 10.1007/s11695-007-9168-1 17894150

[B15] Lemieux I Drapeau V Richard D Bergeron J Marceau P Biron S Waist girth does not predict metabolic complications in severely obese men Diabetes Care 2006 29 1417 1419 10.2337/dc06-0441 16732038

[B16] Ledoux S Coupaye M Essig M Msika S Roy C Queguiner I Traditional anthropometric parameters still predict metabolic disorders in women with severe obesity Obesity 2010 18 1026 1032 10.1038/oby.2009.349 19851304

[B17] Engeland A Bjorge T Selmer RM Tverdal A Height and body mass index in relation to total mortality Epidemiology 2003 14 293 299 10.1097/00001648-200305000-00008 12859029

[B18] Sjostrom LV Mortality of severely obese subjects American Journal of Clinical Nutrition 1992 55 Suppl 523S 10.1093/ajcn/55.2.516s 1531097

[B19] Livingston EH Ko CY Effect of diabetes and hypertension on obesity-related mortality Surgery 2005 137 16 25 10.1016/j.surg.2004.05.049 15614276

[B20] Melanson KJ McInnis KJ Rippe JM Blackburn G Wilson PF Obesity and cardiovascular disease risk: research update. [Review] [50 refs] Cardiology in Review 2001 9 202 207 10.1097/00045415-200107000-00005 11405900

[B21] Claudi T Midthjell K Holmen J Fougner K Kruger O Wiseth R Cardiovascular disease and risk factors in persons with type 2 diabetes diagnosed in a large population screening: the Nord-Trondelag Diabetes Study, Norway Journal of Internal Medicine 2000 248 492 500 10.1046/j.1365-2796.2000.00759.x 11155142

[B22] Cassidy AE Bielak LF Zhou Y Sheedy PF Turner ST Breen JF Progression of subclinical coronary atherosclerosis: does obesity make a difference? Circulation 2005 111 1877 1882 10.1161/01.CIR.0000161820.40494.5D 15837939

[B23] Lakka TA Lakka HM Salonen R Kaplan GA Salonen JT Abdominal obesity is associated with accelerated progression of carotid atherosclerosis in men Atherosclerosis 2001 154 497 504 10.1016/S0021-9150(00)00514-1 11166785

[B24] Laurent S Boutouyrie P Asmar R Gautier I Laloux B Guize L Aortic stiffness is an independent predictor of all-cause and cardiovascular mortality in hypertensive patients Hypertension 2001 37 1236 1241 11358934 10.1161/01.hyp.37.5.1236

[B25] Laurent S Katsahian S Fassot C Tropeano AI Gautier I Laloux B Aortic stiffness is an independent predictor of fatal stroke in essential hypertension Stroke 2003 34 1203 1206 10.1161/01.STR.0000065428.03209.64 12677025

[B26] Vlachopoulos C Aznaouridis K Stefanadis C Prediction of cardiovascular events and all-cause mortality with arterial stiffness: a systematic review and meta-analysis. [Review] [49 refs] Journal of the American College of Cardiology 2010 55 1318 1327 10.1016/j.jacc.2009.10.061 20338492

[B27] Lim HE Park CG Shin SH Ahn JC Seo HS Oh DJ Aortic pulse wave velocity as an independent marker of coronary artery disease Blood Pressure 2004 13 369 375 10.1080/08037050410004800 15771222

[B28] World Medical Association declaration of Helsinki. Recommendations guiding physicians in biomedical research involving human subjects JAMA 1997 277 925 926 10.1001/jama.277.11.925 9062334

[B29] Laurent S Cockcroft J Van BL Boutouyrie P Giannattasio C Hayoz D Expert consensus document on arterial stiffness: methodological issues and clinical applications European Heart Journal 2006 27 2588 2605 10.1093/eurheartj/ehl254 17000623

[B30] Laurent S Boutouyrie P Arterial stiffness: a new surrogate end point for cardiovascular disease?. [Review] [29 refs] Journal of Nephrology 2007 20 Suppl 50 18050143

[B31] Mansia G De BG Dominiczak A Cifkova R Fagard R Germano G 2007 ESH-ESC Guidelines for the management of arterial hypertension: the task force for the management of arterial hypertension of the European Society of Hypertension (ESH) and of the European Society of Cardiology (ESC) Blood Pressure 2007 16 135 232 10.1080/08037050701461084 17846925

[B32] Willum-Hansen T Staessen JA Torp-Pedersen C Rasmussen S Thijs L Ibsen H Prognostic value of aortic pulse wave velocity as index of arterial stiffness in the general population Circulation 2006 113 664 670 10.1161/CIRCULATIONAHA.105.579342 16461839

[B33] Matthews DR Hosker JP Rudenski AS Naylor BA Treacher DF Turner RC Homeostasis model assessment: insulin resistance and beta-cell function from fasting plasma glucose and insulin concentrations in man Diabetologia 1985 28 412 419 10.1007/BF00280883 3899825

[B34] Friedewald WT Levy RI Fredrickson DS Estimation of the concentration of low-density lipoprotein cholesterol in plasma, without use of the preparative ultracentrifuge Clinical Chemistry 1972 18 499 502 4337382

[B35] Zebekakis PE Nawrot T Thijs L Balkestein EJ Heijden-Spek J Van Bortel LM Obesity is associated with increased arterial stiffness from adolescence until old age Journal of Hypertension 2005 23 1839 1846 10.1097/01.hjh.0000179511.93889.e9 16148607

[B36] Czernichow S Bertrais S Oppert JM Galan P Blacher J Ducimetiere P Body composition and fat repartition in relation to structure and function of large arteries in middle-aged adults (the SU.VI.MAX study) International Journal of Obesity 2005 29 826 832 10.1038/sj.ijo.0802986 15917850

[B37] Faintuch J Marques PC Bortolotto LA Faintuch JJ Cecconello I Systemic inflammation and cardiovascular risk factors: are morbidly obese subjects different? Obesity Surgery 2008 18 854 862 10.1007/s11695-008-9504-0 18392896

[B38] Wildman RP Mackey RH Bostom A Thompson T Sutton-Tyrrell K Measures of obesity are associated with vascular stiffness in young and older adults Hypertension 2003 42 468 473 10.1161/01.HYP.0000090360.78539.CD 12953016

[B39] Rider OJ Tayal U Francis JM Ali MK Robinson MR Byrne JP The effect of obesity and weight loss on aortic pulse wave velocity as assessed by magnetic resonance imaging Obesity 2010 18 2311 2316 10.1038/oby.2010.64 20360756

[B40] Anand SS Islam S Rosengren A Franzosi MG Steyn K Yusufali AH Risk factors for myocardial infarction in women and men: insights from the INTERHEART study European Heart Journal 2008 29 932 940 10.1093/eurheartj/ehn018 18334475

[B41] Nordhamn K Sodergren E Olsson E Karlstrom B Vessby B Berglund L Reliability of anthropometric measurements in overweight and lean subjects: consequences for correlations between anthropometric and other variables International Journal of Obesity & Related Metabolic Disorders: Journal of the International Association for the Study of Obesity 2000 24 652 657 10.1038/sj.ijo.0801216 10849590

[B42] Lee CM Huxley RR Wildman RP Woodward M Indices of abdominal obesity are better discriminators of cardiovascular risk factors than BMI: a meta-analysis. [Review] [29 refs] Journal of Clinical Epidemiology 2008 61 646 653 10.1016/j.jclinepi.2007.08.012 18359190

[B43] Huxley R Mendis S Zheleznyakov E Reddy S Chan J Body mass index, waist circumference and waist:hip ratio as predictors of cardiovascular risk--a review of the literature. [Review] [23 refs] European Journal of Clinical Nutrition 2010 64 16 22 10.1038/ejcn.2009.68 19654593

[B44] Webb DR Khunti K Silverman R Gray LJ Srinivasan B Lacy PS Impact of metabolic indices on central artery stiffness: independent association of insulin resistance and glucose with aortic pulse wave velocity Diabetologia 2010 53 1190 1198 10.1007/s00125-010-1689-9 20213236

[B45] Aronson D Cross-linking of glycated collagen in the pathogenesis of arterial and myocardial stiffening of aging and diabetes. [Review] [81 refs] Journal of Hypertension 2003 21 3 12 10.1097/00004872-200301000-00002 12544424

[B46] Hemmingsson E Udden J Neovius M No apparent progress in bioelectrical impedance accuracy: validation against metabolic risk and DXA Obesity 2009 17 183 187 10.1038/oby.2008.474 18997678

[B47] Volgyi E Tylavsky FA Lyytikainen A Suominen H Alen M Cheng S Assessing body composition with DXA and bioimpedance: effects of obesity, physical activity, and age Obesity 2008 16 700 705 10.1038/oby.2007.94 18239555

[B48] Heath EM Adams TD Daines MM Hunt SC Bioelectric impedance and hydrostatic weighing with and without head submersion in persons who are morbidly obese Journal of the American Dietetic Association 1998 98 869 875 10.1016/S0002-8223(98)00201-6 9710656

